# Chemotherapy resistance in metastatic breast cancer: the evolving role of ixabepilone

**DOI:** 10.1186/bcr2573

**Published:** 2010-10-22

**Authors:** Edgardo Rivera, Henry Gomez

**Affiliations:** 1 The Methodist Hospital/Weill Cornell University, 6550 Fannin Street, SM701, Houston, TX 77030, USA; 2 Department of Medical Oncology, Instituto Nacional de Enfermedades Neoplásicas, Av. Angamos Este 2520, Lima, Perú

## Abstract

Resistance to chemotherapy is a major obstacle to the effective treatment of many tumor types. Although many anticancer therapies can alter tumor growth, in most cases the effect is not long lasting. Consequently, there is a significant need for new agents with low susceptibility to common drug resistance mechanisms in order to improve response rates and potentially extend survival. Approximately 30% of the women diagnosed with early-stage disease in turn progress to metastatic breast cancer, for which therapeutic options are limited. Current recommendations for first-line chemotherapy include anthracycline-based regimens and taxanes (paclitaxel and docetaxel). They typically give response rates of 30 to 70% but the responses are often not durable, with a time to progression of 6 to 10 months. Patients with progression or resistance may be administered capecitabine, gemcitabine, vinorelbine, albumin-bound paclitaxel, or ixabepilone, while other drugs are being evaluated. Response rates in this setting tend to be low (20 to 30%); the median duration of responses is <6 months and the results do not always translate into improved long-term outcomes. The present article reviews treatment options in taxane-resistant metastatic breast cancer and the role of ixabepilone in this setting.

## Introduction

The development of chemotherapy resistance continues to be the main problem in the treatment of cancer patients. Newer agents, whether chemotherapeutic or targeted, are constantly being developed. Although most anticancer therapies will alter tumor growth, in most cases the effect is not long lasting and failure of anthracyclines and taxanes impact the survival of breast cancer patients. Consequently, there is a significant need for new agents with low susceptibility to common drug resistance mechanisms in order to improve response rates and potentially extend survival.

Approximately 30% of the women diagnosed with early-stage disease in turn progress to metastatic breast cancer (MBC), for which therapeutic options are limited [1]. After treatment with anthracycline or taxane-based [2] chemotherapy, options are limited as responses are generally low. Response rates range from 30% to 70% but the responses are often not durable, with a time to progression of 6 to 10 months [1,3]. Patients with progression or resistance may be administered capecitabine, gemcitabine, vinorelbine, or albumin-bound paclitaxel, with capecitabine being the only one approved by the US Food and Drug Administration (FDA) after anthracyclines and taxanes [4,5]. Response rates in this setting tend to be low (20 to 30%); the median duration of responses is <6 months [6] and the results do not always translate into improved long-term outcomes.

Resistance to chemotherapy can occur prior to drug treatment (primary or innate resistance) or may develop over time following exposure (acquired resistance) [7]. Patients with breast cancer who are treated with an anthracycline and/or a taxane commonly develop resistance to one or both of the drugs. In some patients, prolonged exposure to a single chemotherapeutic agent may lead to the development of resistance to multiple other structurally unrelated compounds, known as cross-resistance or multidrug resistance (MDR). In primary resistance, MDR can occur without prior exposure to chemotherapy.

Once resistance to taxanes or anthracyclines occurs, few treatment options exist. Most breast cancer patients with resistant or refractory disease are treated with capecitabine as a single agent or in combination. Approximately 75% of patients treated with capecitabine do not respond, and many responders eventually become resistant [8-10]. Other chemotherapeutics that are used for the treatment of MBC resistant to anthracyclines, taxanes, and capecitabine include gemcitabine and vinorelbine [11]. Response rates with these agents in anthracycline-refractory and taxane-refractory disease range from 16 to 25%, and survival is limited [1,12-14]. Resistance is also an issue for women who have human epidermal growth factor receptor-2 (HER2)-positive breast cancer. The HER2-specific inhibitors trastuzumab and lapatinib have demonstrated efficacy in the metastatic setting [15-17]. Most MBC patients treated with trastuzumab, however, develop resistance within 1 year [18].

Recent research has suggested potential novel therapeutic targets for drug-resistant MBC. Tumor stem cells have been identified in many malignancies, including breast cancer [19,20]. Accumulation of drug resistance mutations in stem cells, coupled with their high-level expression of the ATP-binding cassette (ABC) drug transporters, noncycling state, and enhanced DNA repair, may contribute to the generation of resistance to chemotherapy [21]. The high proliferative potential of such cells could therefore result in the rapid regrowth of resistant tumors. Studies are currently investigating the potential to specifically target breast cancer stem cells using agents that block drug transport or other small-molecule inhibitors [20]. It has been proposed that drug resistance may develop early in tumorigenesis, prior to the onset of well-recognized genotypic changes. Targeting initial events in tumorigenesis may suppress the early development of drug resistance. Novel microtubule inhibitors, such as ixabepilone, show significant activity in MBC and do not exhibit cross-resistance with taxanes or other commonly used chemotherapies; they are therefore potential candidates for the treatment of drug-resistant diseases [22,23].

The aim of the present article is to review the current therapeutic alternatives to treat MBC resistant to taxanes.

## Molecular mechanisms of drug resistance

Chemotherapy resistance can arise through a number of different mechanisms, including alterations in drug pharmacokinetics and metabolism, modification of drug target expression or function (for example, gene amplification/overexpression, overexpression of β-tubulin isotypes, and topoisomerase II mutations), drug compartmentalization in cellular organelles, altered repair of drug-induced DNA damage, changes in apoptotic signaling pathways (for example, mutated p53), and expression of proteins directly affecting cellular drug transport (efflux pumps) (Table 1) [24,25]. The heterogeneity of cancer cells, coupled with their high mutation rate, contributes to rapid selection for drug-resistant clones. The best characterized of these resistance mechanisms are drug efflux pathways.

**Table 1:** Mechanisms of drug resistance in breast cancer [24,25]

Class of resistance	Drug examples
Drug transport/sequestration	ABC transporters: P-glycoprotein, multidrug-resistant protein 1 (breast cancer resistance protein)
Modification of drug target (qualitative and quantitative)	Dihydrofolate reductase, epidermal growth factor receptor; C-KIT mutations; tubulin
DNA repair/genomic instability	Mismatch repair proteins; caspases, PTEN; p27; microsatellite instability, loss of heterozygosity, topoisomerase I, topoisomerase II
Regulators of apoptosis	p53; PTEN; Bcl-2, Bcl-x
Drug metabolism/inactivation	Cytochrome P450; glutathione S-transferase; aldehyde dehydrogenase

ABC, ATP-binding cassette; PTEN, phosphatase and tensin homolog deleted on chromosome 10.

Many transport-mediated drug resistance mechanisms involve the ABC membrane transporter family. The most well-characterized examples of these drug efflux transporters include the P-glycoprotein (P-gp) pump, multidrug-resistant protein-1 (MRP1), and breast cancer resistance protein. These energy-dependent proteins actively pump drugs such as chemotherapeutics out of the cells, thereby reducing their intracellular drug concentration and decreasing the cytotoxicity [7].

### Drug transport/sequestration

Expression of pumps such as P-gp or MRP1 gives tumor cells the ability to evade the chemotherapy drugs, and their role has been evaluated in breast cancer.

P-gp is a 170 kDa glycoprotein encoded by the *MDR1* gene. This ATP-dependent membrane transporter pumps a diverse array of chemotherapeutics across the cell membrane and out of the cells, including anthracyclines, taxanes, vinca alkaloids, epipodophyllotoxins, and antifolates. The normal physiologic role of P-gp is still unknown, but it may serve to protect normal tissues from toxic products and xenobiotics [24]. P-gp expression varies widely in breast cancer, according to the assay method used. A meta-analysis revealed that this protein is expressed in approximately 40% of all breast carcinomas [26], although another study reported values as high as 66% [27]. Exposure to selected chemotherapeutics may increase P-gp expression in breast cancer, as seen in some patients following neoadjuvant chemotherapy [28,29]. In the meta-analysis, prior chemotherapy or hormonal therapy was found to enhance the proportion of P-gp-positive tumors by nearly 1.8-fold. This increased P-gp expression was associated with a threefold increased risk of failure to respond to chemotherapy [26]. The expression of P-gp, therefore, correlated with a poorer outcome in this and other studies [30,31], although other reports did not find such an association [27,32].

MRP1 has been also implicated in MDR. MRP1 belongs to the ABC drug transporter family, included with seven known members (MRP1 to MRP7), which all differ in tissue distribution and drug transport specificity [33]. As determined by RT-PCR, MRP1 is expressed in nearly all breast cancers (and in approximately one-half of normal breast tissues) [25]. This protein confers an MDR phenotype similar to, but distinct from, that associated with P-gp. MRP1 mediates resistance to agents such as vinca alkaloids, anthracyclines, and high-dose methotrexate, but not to paclitaxel or mitoxantrone. Some studies suggest that MRP1 expression correlates with poor survival in patients with early-stage disease who received chemotherapy, although a causal relationship is not clear [34].

Another ABC membrane transporter that may play a role in drug resistance is breast cancer resistance protein, since it is involved with the efflux of various chemotherapeutics such as mitoxantrone, anthracyclines, methotrexate, and topoisomerase I inhibitors [35]. Resistance mediated by breast cancer resistance protein is similar to the pattern seen with P-gp-mediated chemoresistance. This transporter may be a marker for tumor stem cells and appears to protect against hypoxia [36,37].

### Modification of drug target

Microtubules are essential components of the cytoskeleton and mitotic apparatus. They are assembled from α-tubulin and β-tubulin heterodimers, along with other proteins such as microtubule-associated proteins. Microtubule-targeting agents both inhibit microtubule polymerization and destabilize microtubules (such as vinca alkaloids), or they promote their polymerization and stabilization (for example, taxanes) [38]. Paclitaxel is known to bind to βIII-tubulin, which is one of the six known β-tubulin isotypes. Binding disrupts the microtubule dynamics by stabilizing microtubules and inducing microtubule bundles, thereby inhibiting cell division and triggering apoptosis [38].

Altered expression of β-tubulin isotypes is found in many cancer cell lines and xenografts resistant to microtubule inhibitors, and this may be associated with the primary or acquired resistance to tubulin-binding agents observed clinically in many tumors (alterations in tubulin and associated proteins can affect the microtubule structure and function, and have been implicated in drug resistance; see Table 2) [39-47]. *In vitro,* the overexpression of the βIII subunit induces paclitaxel resistance, possibly by decreasing paclitaxel’s binding to βIII-tubulin and disrupting the microtubule dynamics [42]. This phenotype was seen in a leukemia cell line that was resistant to vinblastine, which was also cross-resistant to other vinca alkaloids and paclitaxel [48]. Other studies have also observed altered expression levels of tubulin or βIII isoforms that are associated with taxane resistance [40,41]. Additionally, several β-tubulin mutations have been characterized that result in drug resistance [43-45], which is probably due to alterations affecting the drug-binding sites. Owing to the confounding presence of tubulin pseudogenes, however, the clinical significance of these mutations is unclear [49]. Changes in microtubule-associated proteins, such as microtubule-associated protein-4 and tau, can also affect the microtubule dynamics and modulate sensitivity to taxanes and vincas [46,47].

**Table 2:** Role of β-tubulin in drug resistance

Altered expression of β-tubulin isotypes [41,42]
Overexpression of the βIII-tubulin subunit [40,43]
β-Tubulin mutations affecting microtubule stability and the binding of microtubule inhibitors [44-46]
Changes in microtubule-associated proteins (for example, tau and microtubule-associated protein-4) [47,48]
Post-translational modifications of tubulin (for example, acetylation) [40]

Clinically, βIII overexpression may serve as a surrogate for paclitaxel resistance in advanced breast cancer [50]. In breast cancer patients who are treated with first-line paclitaxel, high βIII-tubulin expression correlated with disease progression [51]; similar results were seen in paclitaxel-resistant ovarian cancer [52].

### DNA repair and cellular damage

In addition to P-gp and β-tubulin alterations, other mechanisms have been implicated in breast cancer drug resistance. Alterations in enzymes that are involved in DNA repair or that affect drug sensitivity can also affect drug resistance. Topoisomerase II is a critical enzyme that is involved in DNA replication and repair, in which reduced topoisomerase II expression or function can contribute to resistance to agents such as anthracyclines and epipodophyllotoxins [7,53]. The loss of DNA-mismatch repair activity – which mediates damage repair from many drugs including alkylating agents, platinum compounds, and anthracyclines – has also been implicated in drug resistance [54]. In breast cancer, altered DNA-mismatch repair is associated with microsatellite instability. The loss of function of the DNA-mismatch repair proteins MSH2 and MLH1 resulted in resistance to the topoisomerase II inhibitors epirubicin, doxorubicin, and mitoxantrone, but not to taxanes [55]. The reduced expression of MLH1, following neoadjuvant chemotherapy for node-positive breast cancer, predicted poor disease-free survival [56], and in a study of sporadic invasive ductal carcinoma it was associated with resistance to the adjuvant cyclophosphamide, methotrexate, and fluorouracil [57]. In general, the loss of heterozygosity or microsatellite instability can contribute to tumor progression and may be associated with resistance to certain regimens, such as epirubicincyclophosphamide-based chemotherapy [58].

### Apoptosis

In addition to DNA-mismatch repair, alterations regulating cellular damage can contribute to drug resistance. The levels of the thiol protease caspase-3, a key mediator of apoptosis, were found to be significantly higher in breast cancer compared with normal tissue [59]. The expression of a caspase-3s splice variant was also higher in breast carcinomas compared with nontumor tissue, and increased levels were correlated with resistance to cyclophosphamide-containing chemotherapy [60].

MDR can arise from a failure of the cells to undergo apoptosis following DNA damage or other cellular injury. Mutations in the *p53* tumor suppressor gene (which regulates apoptosis) are found in most human breast cancer cell lines [61], and certain mutations have been linked to *de novo* resistance to doxorubicin and early relapse in breast cancer [62]. In one study, *p53* mutations were a strong prognostic factor for survival in patients with node-positive breast cancer who were administered adjuvant cyclophosphamide, methotrexate, and fluorouracil, which may therefore predict resistance to such therapy [63]. Alterations in other genes regulating the apoptotic pathway, such as *bcl-2* and *bcl-x,* may also promote resistance to tubulin inhibitors [64]. The tumor suppressors phosphatase and tensin homolog deleted on chromosome 10 and p27 both regulate apoptosis, and the decreased expression of these proteins has been proposed to affect the response to trastuzumab [65] and resistance to chemotherapy [66], respectively.

### Drug inactivation/detoxification

Other enzymes may affect breast cancer drug resistance, including those regulating drug inactivation or detoxification. Isoforms of aldehyde dehydrogenase, such as ALDH1 A1 and ALDH3A1, can catalyze the detoxification of cyclophosphamide and may therefore reduce sensitivity to this agent. Higher levels of ALDH3A1 have been found in breast cancer cells compared with normal tissues [67]. Moreover, the cellular levels of ALDH1 A1 (but not ALDH3A1) were significantly higher in those metastatic tumors that did not respond to cyclophosphamide-based regimens, when compared with tumors that were sensitive. Glutathione and glutathione S-transferase are involved in the detoxification of alkylating agents and cisplatin, so the modulation of their activity might affect the resistance to these compounds [68]. Cytochrome p450 is another enzyme that could be involved in resistance in taxanes. Polymorphisms in CYP3A4 and CYP2C8 associated with greater basal enzymatic activity lead to reduced plasma concentrations of the active drug [69].

## Capecitabine

Capecitabine (fluoropyrimidine carbamate) is rationally designed to generate fluorouracil preferentially in tumor tissue and to mimic continuous infusion of fluorouracil. Capecitabine is hydrolyzed in the liver by the enzyme carboxylesterase to produce 5′-deoxy-5-fluorocytidine, is then deaminated on its pyrimidine ring to produce 5′-deoxy-5-fluorouridine by the enzyme cytidine deaminase, located mainly in hepatic and neoplastic tissue, and finally thymidine phosphorylase produces activation of 5′-deoxy-5-fluorouridine to fluorouracil in tumor cells, thus minimizing systemic exposure to fluorouracil [70].

Nowadays, capecitabine is the agent most evaluated in patients treated with taxanes. Clinical evidence supports the use of capecitabine in patients with MBC who have been previously exposed to taxanes. The first trial to evaluate the efficacy and safety of capecitabine (twice-daily oral 2,510 mg/m^2^/day for 2 weeks followed by a 1-week rest and repeated in 3-week cycles) on 162 patients with paclitaxel refractory MBC observed an overall response rate of 20% (95% confidence interval (CI), 14 to 28%) [71]. Diarrhea (14%) and hand-foot syndrome (10%) were the only treatment-related adverse events that occurred with grade 3 or grade 4 intensity in more than 10% of patients [71]. In a posterior phase II trial with 74 patients, an overall response rate of 26%, a median survival of 12.2 months, a median duration of response of 8.3 months, and a median time to disease progression of 3.2 months were observed [8]. With regard to the safety, treatment was well tolerated and the only grade 3 treatment-related adverse events reported in ≥10% of patients were hand-foot syndrome (22%), diarrhea (16%), and stomatitis (12%) [8]. Other trials have also proven the efficacy of capecitabine [9,10].

## Epothilones

Given the clinical significance of drug resistance found in most tumor cells and the challenges this presents for cancer therapy, new agents with novel mechanisms of action are needed. Epothilones represent a new class of microtubule inhibitors that have shown promising activity in MDR tumor cells, and have therefore been explored for the treatment of drug-resistant MBC.

Epothilones are a family of naturally occurring cytotoxic macrolides that inhibit microtubule function. Epothilones A and B, which are two major fermentation products originally isolated from the broth of the myxobacterium *Sorangium cellulosum,* were found to stabilize polymerized microtubules and therefore to inhibit depolymerization [72,73]. The epothilones are structurally different from paclitaxel and docetaxel and may have a distinct mechanism of action [3]. Structural analyses indicate that epothilones may bind at or near the paclitaxel binding site on the β-tubulin protein [74-76]. In contrast to taxanes, certain epothilone B analogs inhibit those drug-resistant cells that overexpress P-gp -suggesting these compounds may be effective for the treatment of drug-resistant tumors, including those with an MDR phenotype.

### Ixabepilone

One of the most active epothilone analogs is the semisynthetic derivative ixabepilone, which has superior stability and water solubility compared with epothilone B [77]*.* Just as in paclitaxel, ixabepilone results in G_2_/M cell cycle arrest and subsequent apoptosis, yet its median inhibitory concentration value is approximately 1 log lower than this taxane [23]. Low nanomolar concentrations of ixabepilone exert broad antitumor activity in a variety of solid tumor cell lines, including breast carcinoma [22,23]. In contrast to paclitaxel, ixabepilone can bind to multiple isomers of β-tubulin, including the βIII isoform [78].

*In vitro,* ixabepilone inhibits the growth of several drug-resistant cell lines, including some that are resistant to paclitaxel (Table 3) [11,22,23,78-80]. Ixabepilone has low susceptibility to various drug resistance mechanisms, such as MDR overexpression [81], β-tubulin mutations [82], and the overexpression of the βIII-tubulin isotype [22,77,83]. Notably, ixabepilone has shown activity in breast cancers with primary and acquired taxane resistance. Ixabepilone is not a good substrate for MDR and does not strongly induce P-gp expression (possibly because of the relatively flexible structure of this compound), which may in part account for its activity in drug-resistant tumors [11]. Ixabepilone is not only active against paclitaxel-sensitive xenografts, but also demonstrates significant activity with paclitaxel-resistant human tumor models including breast carcinoma, ovarian cancer, and colorectal cancer xenografts [22].

**Table 3:** Preclinical activity of ixabepilone in drug-resistant cancer

Active against numerous drug-resistant tumor cell lines, including human paclitaxel-resistant breast cancer cell lines and xenografts [23,76-78]
Inhibitory activity in breast cancers with primary or acquired resistance [23,78]
Low susceptibility to multiple mechanisms of drug resistance [23,76]
Multidrug resistance overexpression: overexpression of the βIII-tubulin isotype
Poor substrate for multidrug resistance; does not strongly induce P-glycoprotein expression [12]

In addition to showing activity in breast cancer, ixabepilone has also shown activity against a variety of other solid tumors. Antitumor activity was noted in cancers that were heavily pretreated or refractory, including platinum-refractory nonsmall-cell lung cancer [84]. Ixabepilone has demonstrated clinical activity in some patients with tumors that are considered chemotherapy resistant, such as renal cell carcinoma [85] and advanced pancreatic cancer [86]. In light of its activity in breast cancer, and particularly in drug-resistant tumors, the clinical activity of ixabepilone was evaluated in patients with drug-resistant MBC.

As discussed previously, alterations in β-tubulin expression (including the βIII isotype) are associated with clinical resistance to taxanes. In contrast to paclitaxel, ixabepilone can bind to βIII-tubulin-containing microtubules, which are dynamically more unstable than βII-tubulin-based microtubules [78]. In addition, ixabepilone is active in preclinical tumor models that are resistant to paclitaxel due to mutations in β-tubulin [22,43]. Together, these results suggest that ixabepilone is effective for the treatment of breast cancer that is resistant to taxanes and to other agents arising from a variety of mechanisms. Molecular mechanisms of resistance to ixabepilone are still unknown and there have been no studies with a representative number of patients, but is suggested that polymorphisms of the carboxyl terminus of class I β-tubulin could be linked to resistance [87].

## Clinical evidence of efficacy of ixabepilone in drug-resistant metastatic breast cancer

Four key clinical trials of ixabepilone in drug-resistant breast cancer have been conducted, including two studies with single-agent ixabepilone and two studies with ixabepilone combined with capecitabine (Table 4) [88-92]. The results of these studies indicate that ixabepilone is active in patients with a pretreated disease, including tumors resistant to anthracyclines, taxanes, and capecitabine, and in patients with widespread metastatic disease.

**Table 4:** Clinical trials of ixabepilone in drug-resistant metastatic breast cancer

**Study**	**Population**	**Evaluable for efficacy/enrolled**	**Pretreatment characteristics**	**Activity**

**Ixabepilone monotherapy**				
Trial 009, phase II [88]	Resistant to taxane; prior treatment with anthracycline-based regimen^a^	49/49	All had received ≥1 prior taxane-based regimen (31 % had ≥2 regimens); 98% had a taxane-containing regimen as their most recent MBC therapy, and 73% had progressed within 1 month of the last administered taxane dose	ORR 12%; 41 % stable disease Median DOR 10.4 months Median TTP 2.2 months (95% CI, 1.4 to 3.2 months) Median OS 7.9 months (95% CI, 6.1 to 14.5 months)
Trial 081, phase II [89]	Resistant to an anthracycline, a taxane, and capecitabine	113/126	77% with visceral disease in liver and/or lung; 88% had completed ≥2 prior chemotherapy regimens for MBC, 48% had ≥3 lines	ORR 11.5%; 50% stable disease Median DOR 5.7 months (95% CI, 4.4 to 7.3 months) Median PFS 3.1 months (95% CI, 2.7 to 4.2 months) Median OS 8.6 months (95% CI, 6.9 to 11.1 months)
**Ixabepilone/capecitabine combination**				
Trial 031, phase II [90]	Anthracycline-pretreated or resistant and taxane-resistant^b^	50/62	72% had baseline visceral metastases, 43% had ≥2 prior chemotherapy regimens in the metastatic setting for MBC	ORR 30%^c^; 32% stable disease Median time to response 6 weeks (range, 5 to 14 weeks) Median DOR 6.9 months (95% CI, 4.3 to 9.7 months)
Trial 046, phase III [92]	Pretreated with or resistant to anthracyclines and resistant to taxanes^d^	737/752	65% had ≥3 metastatic disease sites; 48% had received ≥1 prior regimen for MBC; 85% had progressed on prior taxane therapy for metastatic disease	ORR 34.7% vs. 14.3% Median DOR 6.4 months vs. 5.6 months Median PFS 5.8 months vs. 4.2 months; hazard ratio = 0.75 (95% CI, 0.64 to 0.88)^e^

MBC, metastatic breast cancer; ORR, overall response rate; DOR, duration of response; TTP, time to progression (months); CI, confidence interval; OS, overall survival; PFS, progression-free survival. ^a^Patients had progressed within 4 months of taxane therapy (6 months, if adjuvant therapy only) and had a taxane as their last chemotherapy regimen. ^b^Patients were ineligible if they had received more than three prior chemotherapy regimens for metastatic disease. ^c^All responders had extensive metastatic disease at baseline. ^d^Resistance to anthracycline and taxane is defined as tumor progression during treatment or within 3 months of the last administered dose in the metastatic setting, or recurrence within 6 months in the neoadjuvant or adjuvant setting. This was subsequently revised to include recurrence within 4 months of the last administered dose in the metastatic setting or 12 months in an adjuvant setting. ^e^*P =* 0.0003.

### Taxane-resistant MBC: Trial 009

Given its activity in taxane-resistant breast cancer models, ixabepilone was clinically evaluated in patients with MBC resistant to taxane therapy. An international, multicenter phase II trial evaluated single-agent ixabepilone in patients with MBC who were previously treated with an anthracycline-based regimen and were resistant to a taxane [88]. Patients were eligible if they had progressed within 4 months of taxane therapy in the metastatic setting (6 months if treated with adjuvant therapy only) and had a taxane as their last chemotherapy regimen. Consequently, these tumors were highly resistant to prior treatment with a microtubule-stabilizing agent. Forty-nine patients were administered ixabepilone 40 mg/m^2^, infused over 3 hours, every 21 days for up to 18 cycles due to progressive disease. The overall response rate (ORR) was the primary endpoint.

Most patients in this study had been treated with at least two prior chemotherapy regimens. All of the patients had received at least one prior taxane-containing regimen (31% had at least two regimens), and 98% of patients had a taxane-containing regimen as their most recent therapy in the metastatic setting. This population was highly refractory because 73% of the patients had progressed within 1 month of their last administered taxane dose.

Of the 49 patients eligible for efficacy analysis, there were six responses (ORR 12%) with a median duration of response of 10.4 months. All of the responders had extensive baseline disease and had failed multiple therapies. An additional 20 patients (41%) had stable disease as their best response. The median time to progression was 2.2 months (95% CI, 1.4 to 3.2 months), and the median survival was 7.9 months (95% CI, 6.1 to 14.5 months). Responses seen with ixabepilone in patients with taxane-resistant MBC confirm its clinical activity in this patient population and support its differential sensitivity to the mechanisms of resistance.

### Anthracycline-resistant, taxane-resistant, and capecitabine-resistant MBC: Trial 081

The largest phase II trial evaluated single-agent ixabepilone in patients with heavily pretreated or locally advanced disease or MBC resistant to the three standard chemotherapeutics in this setting; that is, anthracyclines, taxanes, and capecitabine [89]. Resistance to each drug class was defined as disease progression during therapy for MBC (≤8 weeks of the previous treatment) or disease recurrence within 6 months of adjuvant or neoadjuvant chemotherapy with anthracycline or taxane. Ixabepilone 40 mg/m^2^ was administered as a 3-hour intravenous infusion on day 1 of a 21-day cycle. The primary study end-point was the ORR.

The patients in this study had significant and widespread baseline disease: visceral disease in the liver and/ or lung was present in *77%* of patients, and more than 40% had at least three target lesions. The majority of the patients (88%) had completed at least two prior chemotherapy regimens for MBC, and 48% had at least three therapy lines; 15% and 30% of patients had at least one line of anthracycline therapy and taxane therapy, respectively. All but two treated patients had taxane-resistant disease, while 38% had anthracycline-resistant tumors. Many had failed prior chemotherapy for MBC including vinorelbine (25%), gemcitabine (13%), and trastuzumab for HER2-positive disease (9%).

Of the 126 patients enrolled, 113 were evaluable for a response. As assessed independently, the ORR was 11.5% (all partial responses) with another 50% of the patients achieving stable disease as their best response. Tumor responses were durable, with a median duration of 5.7 months (95% CI, 4.4 to 7.3 months); eight of the 13 responders remained progression free for ≥6 months. The median progression-free survival (PFS) was 3.1 months (95% CI, 2.7 to 4.2 months), and the median overall survival was 8.6 months (95% CI, 6.9 to 11.1 months). Ixabepilone monotherapy was therefore active in patients with difficult-to-treat, advanced, highly refractory breast cancer who had failed to respond to prior chemotherapy. One should note that nine of the 12 responders to ixabepilone had not responded to prior multiple lines of chemotherapy in the metastatic setting, including combination regimens.

### Anthracycline-resistant and taxane-resistant MBC: Trial 031

Given the single-agent activity of ixabepilone in women previously treated with anthracyclines, taxanes, and capecitabine, and the need for more effective second-line MBC regimens, the combination of ixabepilone and capecitabine was evaluated in phase II and phase III trials. In the phase II study, patients previously treated with anthracyclines and taxanes were treated with ixabepilone in addition to capecitabine [90]. Sixty-two patients were administered ixabepilone 40 mg/m^2^, infused over 3 hours on day 1, in addition to capecitabine 2,000 mg/m^2^ on days 1 to 14, both given every 21 days. Patients were ineligible if they had received more than three prior chemotherapy regimens for metastatic disease.

Fifty patients were evaluable for a response: 72% had baseline visceral metastases, and 42% received at least two prior chemotherapy regimens for metastatic disease. Fifteen responses occurred (30% ORR), and stable disease was achieved in 32% of patients. All of the responders had extensive metastatic disease at baseline. The median time to response was 6 weeks (range, 5 to 14 weeks), with most responses occurring by the end of the second cycle. The median duration of response was 6.9 months (95% CI, 4.3 to 9.7 months). Four of the 15 responses occurred in patients with estrogen receptor-negative, progesterone receptor-negative, and HER2-negative (triple-negative) breast cancer, suggesting such a regimen may be effective for patients with this treatment-resistant subtype [91]. These preliminary results indicated that the combination of ixabepilone and capecitabine is active in patients with anthracycline-resistant and taxane-resistant MBC.

### Anthracycline-resistant and taxane-resistant MBC: Trial 046

These encouraging phase II results led to an international, randomized, open-label phase III trial that compared ixabepilone plus capecitabine with solely capecitabine administration in patients with locally advanced or MBC pretreated with or resistant to anthracyclines and taxanes [92]. Patients were treated with ixabepilone 40 mg/m^2^, administered as a 3-hour infusion on day 1 of a 21-day cycle, plus capecitabine 2,000 mg/m^2^ on days 1 to 14 of a 21-day cycle. Those patients on capecitabine alone were administered a dose of 2,500 mg/m^2^ on days 1 to 14 of a 21-day cycle. The primary endpoint was PFS. The patients enrolled in this study (*n =* 752) had widespread disease and were heavily pretreated with chemotherapy. Most patients (65%) had at least three metastatic disease sites, and nearly one-half had received at least two prior regimens for metastatic disease. The majority of patients (85%) had progressed on prior taxane therapy for MBC.

**Figure 1:**
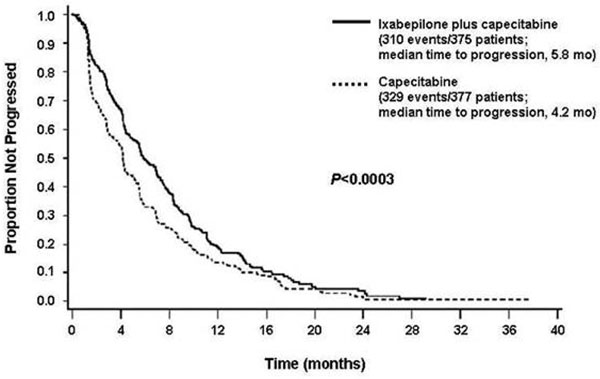
**Progression-free survival for patients treated with ixabepilone plus capecitabine.** Kaplan-Meier progression-free survival curve from a phase III trial of ixabepilone plus capecitabine for metastatic breast cancer patients progressing after anthracycline and taxane treatment [88]. Reprinted with permission from *Journal of Clinical Oncology.*

The trial results demonstrated that PFS significantly improved for patients treated with ixabepilone plus capecitabine compared with capecitabine alone (hazard ratio, 0.75; 95% CI, 0.64 to 0.88; *P =* 0.0003), in turn reflecting a 25% reduction in the estimated risk of disease progression (Figure 1). Median PFS increased by 40% with the combination (5.8 months vs. 4.2 months). Subset analyses indicated that the PFS benefit occurred across subgroups. The ORR also significantly increased in the ixabepilone/capecitabine arm (35%; *P <* 0.0001) compared with capecitabine alone (14%); stable disease occurred in 41% and 46% of patients, respectively. The combination regimen demonstrated activity in triple-negative disease, confirming the activity observed in this subgroup in the phase II trial. Mature overall survival data are anticipated within several months. The most frequent grade 3/4 adverse events in the ixabepilone plus capecitabine group were peripheral sensory neuropathy (with a median onset of four cycles), hand-foot syndrome, fatigue, myalgia, asthenia, and diarrhea; while the most frequent grade 3/4 adverse events in the capecitabine group were hand-foot syndrome and diarrhea, but with incidences similar to those for the combination arm. The incidence of adverse events commonly associated with capecitabine, such as hand-foot syndrome, were not exacerbated by the addition of ixabepilone.

### Other metastatic breast cancer patient populations

In addition to its efficacy in breast cancer resistant to chemotherapy, ixabepilone may also be effective for the treatment of other difficult-to-treat populations. A prospective subset analysis of the above phase III trial evaluated the response in HER2-positive patients who had been pretreated with or were resistant to anthracyclines and taxanes, and who had progressed on trastuzumab [93]. The combination of ixabepilone and capecitabine significantly prolonged median PFS (5.3 months vs. 4.1 months) and the ORR (31% vs. 8%) compared with capecitabine monotherapy, which is similar to the benefit observed in the overall population.

In a phase II trial, ixabepilone was combined with trastuzumab and carboplatin in patients with HER2-positive MBC [94]. Of the 57 patients evaluable for a response, two had complete responses (3.5%), 22 had partial responses (38.6%), and 13 had stable disease for >6 months (22.8%); the median PFS was 8 months. A second prospectively defined subgroup analysis of the phase III study evaluated the combination regimen in patients with anthracycline-pretreated or anthracycline-resistant MBC whose tumors were estrogen receptor-negative [95]. Ixabepilone plus capecitabine resulted in a median PFS of 4.4 months (95% CI, 4.1 to 5.6) versus 2.8 months (95% CI, 2.1 to 3.4) with capecitabine alone, and in a threefold increase of ORR (30% vs. 10%). These data suggest that ixabepilone combined with capecitabine may be effective for the treatment of various MBC patient populations with a poor prognosis and limited treatment options.

## Toxicity

Ixabepilone is associated with a generally manageable safety profile. The toxicities associated with single-agent ixabepilone therapy are usually of a low grade and are comparable with those from other cytotoxic agents commonly used for breast cancer. In the four trials reported in the present review, the most common hematologic toxicity was myelosuppression, primarily neutropenia. Grade 3/4 neutropenia occurred in 53% of patients resistant to taxanes and in 54% of those resistant to anthracyclines, taxanes, and capecitabine. Grade 3/4 leukopenia was observed in 2% of taxane-resistant patients and in 49% of taxane-resistant, anthracycline-resistant, and capecitabine-resistant patients. Febrile neutropenia was rare [88,89]. Similar to other microtubule inhibitors, neuropathy was one of the most frequent treatment-related adverse events occurring with ixabepilone. This was usually mild to moderate in severity and generally resolved after dose adjustments were made. Peripheral sensory neuropathy was the most frequent grade 3/4 treatment-emergent adverse event (12 to 14%). This toxicity was usually reversible, with resolution to grade 1 or baseline within a few weeks in the vast majority of patients. The frequency and severity of this toxicity with ixabepilone was comparable with that observed with other microtubule inhibitors (2 to 32%) [96-99]. The combination of ixabepilone and capecitabine was well tolerated, with minimally overlapping toxicities. Apart from peripheral neuropathy, there was no worsening of capecitabine-associated toxicities (for example, hand-foot syndrome, fatigue, and vomiting) with the combination regimen.

## New drugs and the future of the treatment of metastatic breast cancer resistant to paclitaxel

While ixabepilone is being evaluated in combination with other drugs, new drugs are currently being tested and have the potential to become standard treatments in this MBC setting. Albumin bound paclitaxel (*nab*-paclitaxel) has been studied in a phase II study of weekly albumin-bound paclitaxel for patients with MBC heavily pretreated with taxanes. Response rates were 14% and 16% for the 100 mg/m^2^ and 125 mg/m^2^ cohorts, respectively; an additional 12% and 21% of patients, respectively, had stable disease with an acceptable toxicity profile [100].

Larotaxel is a semisynthetic taxoid that has shown preclinical and clinical activity against taxane-resistant MBC, and has the ability to cross the blood-brain barrier. In a study of larotaxel in combination with trastuzumab in patients with HER2-positive MBC, 42.3% of confirmed partial responses were achieved with a manageable toxicity [101]. Another taxoid currently evaluated in taxane-resistant tumors is cabazitaxel. Although cabazitaxel has not been evaluated in breast cancer, results on a phase III prostate cancer are available [102].

Poly(ADP ribose) polymerase inhibitors are one group of drugs with great potential in resistant breast cancer, especially triple-negative and BRCA-deficient breast cancer. A phase II study of olaparib in confirmed BRCA1/ BRCA2 carriers with advanced refractory breast cancer showed an ORR of 38% [103]. Other poly(ADP ribose) polymerase inhibitors being evaluated include veliparib in combination with temozolamide, results for which will be available in the near future [104].

## Conclusion

Drug resistance (primary or acquired) is a leading cause of treatment failure in patients with cancer, especially MBC. Patients with advanced or MBC commonly develop disease resistance to chemotherapy and even biologic therapies such as trastuzumab, leaving few effective treatment options. The occurrence of MDR disease in many patients with advanced breast cancer due to the overexpression of βIII-tubulin isotype or drug transporters, such as P-gp, demands new approaches. Consequently, there is a significant need for novel agents that are effective in drug-resistant tumors with mechanisms of action that are different from the available chemotherapeutics.

The epothilone B analog ixabepilone demonstrates significant antitumor activity against a variety of tumor cells with primary or acquired drug resistance, including MDR. Ixabepilone is less susceptible to the common mechanisms of drug resistance, particularly tubulin mutations, compared with taxanes and other traditional chemotherapy. Clinical trials demonstrate single-agent ixabepilone to be active in MBC patients with highly resistant or refractory disease who have a significant tumor burden. Antitumor activity was observed in those patients who have had extensive prior therapy with anthracyclines, taxanes, and/or capecitabine. Ixabepilone toxicity was manageable and comparable with other commonly used chemotherapeutics for MBC. In combination regimens, ixabepilone plus capecitabine resulted in greater activity compared with capecitabine alone in a taxane-resistant population, without significantly increasing toxicity. Ixabepilone has been approved by the US Food and Drug Administration for use in combination with capecitabine for the treatment of locally advanced breast cancer or MBC after the failure of an anthracycline and a taxane, and as monotherapy after the failure of an anthracycline, a taxane, and capecitabine. A previous publication suggests that the cost-effectiveness ratio could be higher for addition of ixabepilone to capecitabine treatment [105].

The potential of ixabepilone in patients with early-stage breast cancer is currently under evaluation. Given the clinical impact of drug resistance in breast cancer and other malignancies, new agents are clearly needed with differential sensitivity to the various mechanisms of tumor resistance compared with the standard chemotherapy drugs. Increased application of pharmacogenomics may also allow for the identification of patients with, or at increased risk for, drug resistance as well as those who are most likely to benefit from the treatment.

## Abbreviations

ABC: ATP-binding cassette; CI: confidence interval; HER2: human epidermal growth factor receptor-2; MBC: metastatic breast cancer; MDR: multidrug resistance; MRP1: multidrug-resistant protein-1; ORR: overall response rate; PCR: polymerase chain reaction; PFS: progression-free survival; P-gp: P-glycoprotein; RT: reverse transcription.

## Competing interests

The author receives research grants from Bristol Myers.

Published: 22 October 2010
